# Assessment of Pulmonary Function Parameters, Signs, and Symptoms in the Employee of a Dairy Products Company in Tehran, Iran

**Published:** 2018-01

**Authors:** Mohammad Hassan Nassiri-Kashani, Mashallah Aghilinejad, Amir Bahrami-Ahmadi, Omid Moslemi, Elahe Kabir-Mokamelkhah

**Affiliations:** Occupational Medicine Research Center, Iran University of Medical Sciences, Tehran, Iran

**Keywords:** Dairy Companies, Respiratory Symptoms, Spirometry

## Abstract

**Background:**

Workers in dairy products companies are exposed to different respiratory hazards. The current study aimed at investigating and comparing the prevalence of pulmonary function parameters, signs, and symptoms in the exposed workers and office staff.

**Materials and Methods:**

The current cross sectional study was conducted in 2015 on 864 employees working in a dairy products company in Tehran, Iran. The subjects included 646 employees exposed to respiratory hazards at the production line and the other group consisted of 218 staff not exposed to respiratory hazards in the production line as the unexposed group. Demographic characteristics and the presence of respiratory symptoms and signs were gathered using a checklist. Spirometric indices including FEV_1_, forced volume vital capacity (FVC), and FEV_1_/FVC were measured for the study participants.

**Results:**

Although exposure to the respiratory hazards among participants of the exposed group was lower than permitted limits, the frequency of respiratory signs and symptoms were significantly higher than those of the unexposed staff. In the current study, mean percentage of FVC, FEV_1_/FVC, and FEV_1_ were significantly less than the predicted amount in the exposed group than in the unexposed group.

**Conclusion:**

Although the frequency of respiratory signs and symptoms was lower than those of other similar studies, abnormal spirometric patterns were common; hence, it can be pointed out that even in the work environments, such as dairy products industry with below the permissible exposure limit of respiratory risks, multiple spirometric disorders can be observed. In other words, the absence of respiratory signs and symptoms are insufficient and application of different pulmonary function tests, such as spirometry, seem essential for assessment.

## INTRODUCTION

Employees of different industries are at risk of various respiratory diseases, due to their working environment and types of their jobs. Several studies reported that some of these risk factors can have short- or even long-term impacts on the exposed workers and lead to respiratory signs and symptoms, different spirometric abnormalities, and decreased pulmonary function in such individuals (1). In the study by Aghilinejad et al., on stone workers exposed to silica dust, the prevalence of respiratory symptoms was significantly higher than those of other employees, even in workers with normal spirometric pattern (2). Occupational specialists try to assess the association between different risk factors and their impacts on certain jobs tasks. In other words, these researchers try to develop a preventive program to reduce the impact of risk factors on the exposed employees (3, 4).

Most studies showed that exposure to higher-than-standard amounts can cause respiratory signs and symptoms and reduce the pulmonary function in the employees of various industries (5–8). It seems that exposure to respiratory risk factors, even in a low-limit amount, can cause respiratory symptoms and change the spirometric indices in the exposed workers.

Accordingly, the current study aimed at investigating and comparing the prevalence of pulmonary function parameters, signs, and symptoms in the exposed workers and office staff.

## MATERIALS AND METHODS

The current cross sectional, observational, analytical study was conducted on 864 employees working in a dairy products company in Tehran, Iran. The study protocol was approved by the Ethics Committee of Occupational Medicine Research Center, Iran University of Medical Sciences, Tehran, Iran. According to the reports of health professionals, all respiratory exposures in the work environment of this production unit, including acid and alkaline vapors, metal fumes, and solvent vapors were measured and the amounts below the permissible exposure limit were recorded in the annual reports of occupational health. According to the study protocol, the participants in the study were divided into two groups. The exposed workers included 646 employees with at least one year experience of working in the production line, exposed to the risk factors of respiratory diseases listed above, and the other group consisted of 218 staff with more than one year of work experience, not exposed to respiratory risks in the production line. Among the study participants, workers with proven pulmonary disease, the ones with a second job, or a history of working in the environments with respiratory risk factors were excluded from the study.

### Assessing the respiratory function and physical examination

The first part of the checklist, completed for all participants, included demographic information (age, gender, body mass index (BMI), marital status, and smoking). The participants were asked about the presence of respiratory symptoms, including exertional dyspnea, cough, and sputum. Then, they were visited by a pulmonary specialist and underwent a thorough physical examination and lung auscultation, and were assessed for respiratory signs, including wheezing, crackles, and rhonchi.

### Spirometry

Two trained technicians performed the spirometry test for all the participants in the current study using a standard spirometers device (SPM 300) under the surveillance of a pulmonary specialist who supervised the spirometry process, and quality of the results, and confirmed the protocol and results. In the current study, spirometric indices including FEV_1_, forced volume vital capacity (FVC), and FEV_1_/FVC measured for the study participants.

### Statistical Analysis

After collecting the questionnaires, the data were transferred into SPSS and analyzed. Mean and standard deviation (SD) were used to express the quantitative data and frequency and percentage to indicate the qualitative data. To compare the quantitative variables between the groups, the independent student *t* test was employed, while chi-square test was used to compare the qualitative variables. P <0.05 was considered the level of significance.

## RESULTS

In the current study, the respiratory signs and symptoms and spirometric patterns of 446 exposed workers of production line, employed in a dairy products company, were compared with those of 218 unexposed staff, working in the offices of the same company. In our study, 314 participants (70.40%) were male and 132 (29.60%) female. The mean age and mean work experience of participants were 38.32±6.83 and 7.62±2.46 years, respectively. In the current study, mean age of participants in the exposed and unexposed groups were 35.12±8.21 and 36.24±6.36 years, respectively with no significant difference between the groups (P=0.08). In the current study, the mean work experience of the exposed employees was 7.51±3.25 years and that of unexposed group was 8.12±5.31 years with no significant difference between the groups (P=0.8).

Although the unexposed staff had a higher BMI than the exposed workers, there was no significant difference between the two groups (25.25±4.81 vs. 24.62±4.16 kg/m^2^; P=0.14); 86 subjects (19.28%) in the exposed group and 48 (22.2%) in the unexposed group were smokers; there were no significant differences in the smoking rate between the two groups (P = 0.68). In addition, the frequency of smoking did not differ significantly between the two groups (5.6±4.6 vs. 6.6±4.4 pack/year; P=0.23)

The prevalence of all respiratory signs and symptoms in the exposed workers was more than those of office staff, with significant differences in cough (P<0.001), dyspnea (P<0.001), and wheezing (P=0.03) (Table 1).

**Table 1.** Comparison of the frequency of respiratory symptoms in the study groups of exposed and unexposed workers

The study group	Exposed (N = 446)	Unexposed (N = 218)	P-value

Respiratory signs and symptoms	Number (%)	Number (%)	
Cough	81 (18.16)	22 (10.09)	P<0.001
Sputum	45 (10.08)	20 (9.17)	0.14
Dyspnea	54 (12.11)	11 (5.05)	P<0.001
Wheezing	58 (13.01)	16 (7.34)	0.03
Crackle	17 (3.81)	7 (3.21)	0.07
Rhonchi	25 (5.61)	5 (2.29)	0.06

The mean FEV_1_ percentage, compared with the predicted amount was significantly lower in the exposed group than in the unexposed group (78.5±19.2 vs. 107.5±22.7; P<0.001). Moreover, the amount of FVC index was significantly lower in the exposed group than the unexposed group (76.4±12.2 vs. 102.28±30.39) (P<0.001); the FEV_1_/FVC ratio was significantly lower in the exposed group than the unexposed group (92.3 ±13.4 vs. 83.5 ± 6.07; P<0.001). The peak expiratory flow (PEF) index was not significantly different between the exposed and unexposed groups (82.12±17.02 vs. 83.8±19.6; P=0.07). In the current study, different spirometry patterns were determined based on spirometric indices of the employees; restrictive and obstructive patterns were significantly more in the exposed workers than the unexposed ones. But, this difference was not significant in the mixed pattern between the two groups (Table 2).

**Table 2.** Comparison of spirometric patterns in the study groups of exposed and unexposed workers

The study group	Exposed (N = 446)	No exposure (N = 218)	P-value

Spirometric patterns	Number (%)	Number (%)	
Restrictive	85 (19.06)	11 (5.05)	P<0.001
Obstructive	94 (21.08)	16 (7.34)	P<0.001
Mixed	14 (3.14)	5 (2.29)	0.54
Normal	253 (56.72)	186 (85.32)	P<0.001

## DISCUSSION

Results of the present study showed that in the exposed workers of dairy products company, exposure to respiratory rates was lower than permissible exposure limits, but the frequency of respiratory signs and symptoms was higher than those of the unexposed workers. Most of the similar studies reported that the prevalence of respiratory symptoms was significantly higher in the exposed workers than the unexposed ones. Activity in dairy products companies was in most cases associated with symptoms of respiratory disorders, particularly bronchial stenosis and reduced respiratory function (9, 10).

In the current study, although acute and chronic symptoms of respiratory disorders were not differentiated, the obtained results showed that the prevalence of either acute or chronic symptoms was higher among the exposed workers than unexposed employees. However, the majority of similar studies conducted so far focused on the signs and symptoms of chronic respiratory symptoms (11, 12), while there are studies reporting acute respiratory symptoms in workers of various industries (13, 14). In the current study, the most common respiratory symptom was cough with a prevalence of 18.16%. Apparently, the different risk factors in the workplace in the exposed workers had a major role in the significant differences in the frequency of cough and general respiratory symptoms in this group of participants.

In the current study, more than half of the studied employees (56.72%) in the exposed group had normal spirometry pattern, and obstructive pattern was the most prevalent abnormal spirometry pattern in the participants, with a frequency of 21.8%, followed by restrictive pattern with a frequency of 19.6%; similar spirometry patterns were also reported in other studies (15–17). According to the estimates by health professionals in the workplace, it seems that different respiratory risk factors in the workplace can cause respiratory problems and obstructive or restrictive spirometry patterns in the studied employees. In some other similar studies, the frequency of restrictive spirometry patterns was reported more than that of the obstructive pattern; when the study was controlled for all confounding factors such as different underlying diseases, tester error, and observer error, it seemed that the difference in the prevalence was due to work issues such as exposure to respiratory risk factors in the production line (16–18). In our study, both obstructive and restrictive patterns were common and it can be concluded that in this production line, there were several risks with different mechanisms that can cause obstructive patterns and, in some cases, restrictive patterns in the participants.

## CONCLUSION

In the current study, although the frequency of respiratory signs and symptoms was lower than those of other similar studies, abnormal spirometry patterns were common; hence, it can be pointed out that even in work environments such as dairy products industry, with a below the permissible exposure limit of respiratory risks, multiple spirometry disorders can be detected. In other words, the absence of respiratory signs and symptoms are insufficient and different pulmonary function tests, such as spirometry, seem essential for assessment.
